# Leaders in Social Networks, the *Delicious* Case

**DOI:** 10.1371/journal.pone.0021202

**Published:** 2011-06-27

**Authors:** Linyuan Lü, Yi-Cheng Zhang, Chi Ho Yeung, Tao Zhou

**Affiliations:** 1 Research Center for Complex System Science, University of Shanghai for Science and Technology, Shanghai, People's Republic of China; 2 Web Sciences Center, University of Electronic Science and Technology of China, Chengdu, People's Republic of China; 3 Department of Physics, University of Fribourg, Chemin du Musée 3, Fribourg, Switzerland; Universita' del Piemonte Orientale, Italy

## Abstract

Finding pertinent information is not limited to search engines. Online communities can amplify the influence of a small number of power users for the benefit of all other users. Users' information foraging in depth and breadth can be greatly enhanced by choosing suitable leaders. For instance in delicious.com, users subscribe to leaders' collection which lead to a deeper and wider reach not achievable with search engines. To consolidate such collective search, it is essential to utilize the leadership topology and identify influential users. Google's PageRank, as a successful search algorithm in the World Wide Web, turns out to be less effective in networks of people. We thus devise an adaptive and parameter-free algorithm, the LeaderRank, to quantify user influence. We show that LeaderRank outperforms PageRank in terms of ranking effectiveness, as well as robustness against manipulations and noisy data. These results suggest that leaders who are aware of their clout may reinforce the development of social networks, and thus the power of collective search.

## Introduction

Many social networks such as twitter.com and delicious.com allow millions of users to interact, among which some members hold much larger influence than the others. Identifying these influential users is not easy, yet it is essential to identify them: what an online community can collectively achieve is to enhance the power of individuals in discovering new information in depth and breadth that no individual can even contemplate, and an effective way is to make use of influential users. We take the World Wide Web as an example. Though many useful pages are out there, the sheer size of WWW creates a great barrier for comprehensive information exploration. Besides search engines, there is another mode of information acquisition through leveraging the network power, getting useful webpages from different experts. This collective search [1], [2] may one day complement the current search paradigm based on isolated queries, and the key to its success is to identify influential users in social communities.

To identity influential users, we examine delicious.com, a representative online social network. The primary function of delicious.com for individuals is to collect useful bookmarks, such that specific bookmarks can be easily recalled among thousands of them. But for many users, its new function of networking people is more interesting. In delicious.com, users can select other users to be their *leaders*, in the sense that the bookmarks of the leaders are often useful and subscriptions to these bookmarks will be automatic. The subscribers, which we call *fans*, can in turn be the leaders of other users. These relations between leaders and fans connect about half a million of delicious users, forming a *leadership network*. To quantify individual influence, the complex structure and topology of the leadership network embody the non-trivial yet essential information.

Although this leadership network is highly informative for leader identification, to well utilize the network is challenging [3]–[7]. First of all, the leadership structure is complex and going upstream by indefinitely climbing up the ladder of leaders is not illuminating. In addition, considering only the leaders alone provides no absolute measure of influence, as it is the entire upstream connection which act as the information sources and contribute to the influence of a user. Similarly, as we shall see in our experiments, merely counting the number of fans is not a good way to quantify the leader significance. A sophisticated model however could reveal the intrinsic structure and identify the worthy leaders.

To well utilize the leadership network we shall devise a method akin to *PageRank* [8], [9], which effectively ranks webpages based on the hyperlink network. However, the leadership network is fundamentally different as personal relationships are quickly evolving, which makes adaptability essential for ranking users. For instance, the probability which describes the random information acquisition should self-adjust when users add or remove leaders. While this probability is governed by an external parameter in PageRank, we devise our *LeaderRank* algorithm where this probability is adaptive and personalized, leading to a parameter-free algorithm readily applicable to any type of graph. This advantage eliminates the frequent needs of parameter tests and calibration of PageRank on fast evolving networks. Simulations show that our LeaderRank algorithm outperforms PageRank in identifying users who lead to quick and wide spreading of useful items. Moreover, LeaderRank is more tolerant of noisy data and robust against manipulations.

In addition to ranking, the present study may shed light on the future design of community rules and online social networks. Leader identification reinforces well-placed individuals to go deeper and wider in information exploration, where the whole society benefits from the collective outputs. A robust ranking algorithm also discourages people from manipulations [10]. In this paper, we will compare ranking based on the leadership network with simple ranking based on the number of fans. By conducting simulations and experiments, we will see how ranking algorithms identify influential users in social networks. Interested readers may try the webpage http://rank.sesamr.com, where we implement LeaderRank to rank users in delicious.com.

## Materials and Methods

In many online applications, users are able to select other users to be their sources of information. We represent these user-user relations by a network with directed links pointing from fans to their leaders. The link direction corresponds to votes from fans for their leaders, and popular leaders would have a large number of in-links. We take this convention as it matches the direction of random walk in our algorithm, but one may note that the direction of information flow in the network is *opposite*, i.e. from leaders to fans. Our aim is to rank all the users based on this network topology.

### LeaderRank

**Figure 1:**
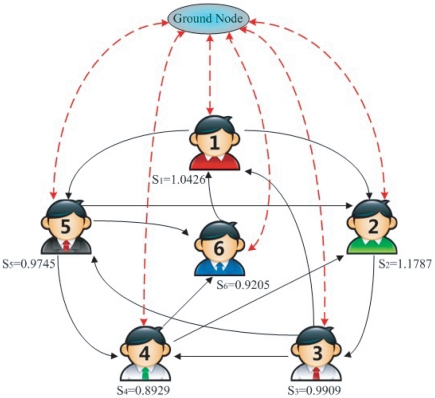
An illustration of the ground node and the LeaderRank algorithm. The social network consists of six users and 12 directed links. The final ranking scores are labeled next to the corresponding users.

We consider a network of 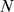 nodes and 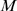 directed links. Nodes correspond to users and links are established according to the relations among leaders and fans. To rank the users, we introduce a *ground node* which connects to every user through bidirectional links (see Fig. 1 for an illustration). The network thus becomes strongly connected and consists of 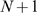 nodes and 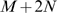 links. To start the ranking process, we assign to each node, except for the ground node, one unit of resource which is then evenly distributed to the node's neighbors through the directed links. The process continues until steady state is attained. Mathematically, this process is equivalent to random walk on the directed network, and is described by the stochastic matrix 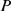 with elements 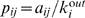 representing the probability that a random walker at 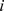 goes to 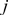 in the next step. 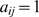 if node 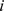 points to 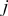 and 0 otherwise, while 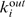 denotes the out-degree, i.e. the number of leaders, of 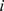. This probability flow thus corresponds to the vote from fan 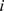 to leader 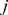. Denoting by 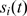 the score of node 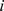 at time 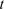, we have
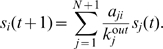
(1)The initial scores are given by 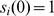 for all node 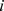 (other than the ground node) and 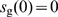 for the ground node.

The presence of the ground node makes 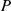 irreducible, as the network is strongly connected. The ground node also ensures the co-existence of loops of size 2 and 3 from any node, which implies 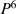 is positive, i.e. all elements of 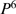 are greater than zero. As 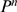 is positive for some natural number 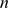, the non-negative 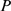 is primitive. By the Perron-Frobenius theorem, 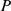 has the maximum eigenvalue 1 with an unique eigenvector. We outline the proof of primitivity and convergence in Text S1 of the *Supporting Information* (*SI*). The score 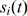 for all 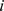 thus converges to a unique steady state denoted as 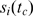, where 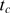 is the convergence time. At the steady state, we evenly distribute the score of the ground node to all other nodes to conserve scores on the nodes of interest. Thus we define the final score of a user to be the leadership score 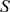, namely
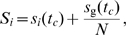
(2)where 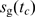 is the score of the ground node at steady state. Based on the above properties, there are several advantages of applying LeaderRank in ranking, which include: (i) parameter-freeness, (ii) wide applicability to any type of graph, (iii) convergence to an unique ranking, and (iv) independence of the initial conditions. For interested readers, we attached the source code of LeaderRank in the final section of Text S1 of SI.

To illustrate the ranking process, we provide a simple ranking example in Fig. 1. After convergence, the final scores of the six users are 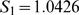, 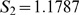, 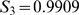, 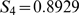, 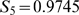 and 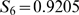, respectively. Therefore, user 2 is ranked top by the LeaderRank algorithm.

### PageRank

We briefly describe the PageRank algorithm, with which we compare our ranking results. PageRank forms the basis of the Google search engine and represents a random walk on the hyperlink network. A parameter 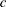 is introduced as the probability for a web surfer to jump to a random website and 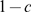 is the probability for the web surfer to continue browsing through hyperlinks. 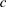 is thus called the *return probability*, i.e. the probability that the web surfer returns and starts a new random walk. In this case, 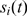 of a webpage 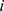 at time 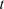 is given by
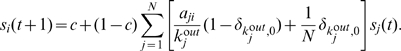
(3)where 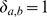 when 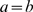 and 0 otherwise. The first and second term respectively correspond to the contributions from random surfers and from surfers arriving through hyperlinks.

Before comparing the ranking results, there are several drawbacks in applying PageRank to social networks. Firstly, return probability is essential in PageRank [8], [9] as algorithmic convergence is only guaranteed on strongly connected networks. This introduces a parameter to the algorithm, and results in the frequent need of extensive tests on parameter and evaluation metrics, which makes PageRank maladaptive to the fast evolving social networks. In addition, return probability is identical for all users irrespective of their significance. For dangling users (those without leaders), specific treatments are required to distribute all their probability back to the network uniformly [8]. All these drawbacks limit the potential of applying PageRank to rank users in social networks, as well as other ranking tasks.

### Differences between LeaderRank and PageRank

An obvious difference between LeaderRank and PageRank lies in the formulation, where the ground node in LeaderRank plays an important role in regulating probability flows, making LeaderRank parameter-free. An essential difference lies in the heart of dynamics, as in LeaderRank the score flow to the ground node is inversely proportional to the number of selected leaders, while there is no such relation in PageRank. We show in Fig. S1 a comparison between the score flow to the ground node with the score flow to random nodes in PageRank. A possible empirical analogy of these score flows is shown in Fig. S2. Mathematically, the score flow to the ground node is analogous to the return probability in PageRank, and the dependence of score flow on the number of leaders makes LeaderRank adaptive to fast evolving networks. The inverse proportion is reasonable, as nodes with a small number of leaders receive less information and hence acquire more information from the ground node (which corresponds to a larger score flow to the ground node). The same happens on the Internet, as web surfers surfing on websites with small out-degree have limited choices of hyperlink and by higher chance jump to another random website. More detailed discussions are given in the first section of Text S1 of SI.

### Data description

We apply the LeaderRank algorithm on the leadership network obtained from the world-largest online bookmarking website, delicious.com, to rank users according to their importance. Users in delicious.com are allowed to collect URLs as bookmarks, and are encouraged to select a list of leaders as sources of information. The dataset we are going to test was collected at May 2008, which consists of 582377 users and 1686131 directed links. Out of which 571686 users belong to the giant component, while the total users in other components are less than 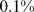 of the giant component. Actually, the numbers of users in the second to fifth largest components are respectively 58, 53, 44 and 35. We thus study only the largest component. The number of directed links in the largest component is 1675008, of which 338756 links (169378 pairs) are reciprocal. If the network is considered as an undirected network, the clustering coefficient [11] and assortativity coefficient [12] are respectively 0.241 and −0.012, while the average shortest distance between users is approximately 5.104.

## Results

We first show the difference among the rankings obtained by LeaderRank, PageRank and the number of fans. Table 1 shows the top 20 users ranked by the three approaches. To have a preliminary evaluation of these ranking results, we compare the ranks with intrinsic qualities of the users which are independent of the ranking algorithm. Specifically, we compare the number of saved bookmarks which may represent the activity of users. In particular, the users *blackbelfjones*, *regine*, *zephoria* and *djakes* who appear in the top 20 of LeaderRank but not in PageRank have activity 5925, 6711, 1486 and 5082 respectively, compared to the smaller activity 3, 377, 1516 and 242 of the users *thetechguy*, *cffcoach*, *samoore* and *kevinrose* who appear in the top 20 of PageRank but not in LeaderRank. This suggests that LeaderRank outperforms PageRank in identifying active users.

**Table 1:** Top 20 users ranked by the three approaches.

User ID	Ranking		
	LeaderRank	PageRank	By the number of fans
adobe	1	1	1
twit	2	2	2
wfryer	3	6	3
willrich	4	7	4
joshua	5	8	6
cshirky	6	12	13
hrheingold	7	15	12
ewan.mcintosh	8	14	19
dwarlick	9	19	14
twitarmy	10	3	
merlinmann	11	16	5
blackbeltjones	12		
jdehaan	13	9	
regine	14		9
lseymour	15	10	
jonhicks	16	17	10
zephoria	17		15
isola	18	11	
djakes	19		
secondlife	20	13	
thetechguy		4	
cffcoach		5	
samoore		18	
kevinrose		20	11
steverubel			7
jgwalls			8
ambermac			16
jgates513			17
ramitsethi			18
cory  arcangel			20

More detailed results and the corresponding discussions are given in Text S1 of *SI*. For instance, the table of the top 100 users is given in Table S1 of *SI*. We have also examined the relation between scores and ranks for all the approaches, where Zipf's laws are observed and shown in Fig. S3 of *SI*. The overlap among the rankings obtained by LeaderRank, PageRank and the number of fans is shown in Fig. S4 of *SI*. By comparing the relationship between the rank and the number of leaders (given in Fig. S5 of *SI*), we find that PageRank tends to assign high rank to nodes with small number of leaders. It is unfair to nodes with large number of leaders, as users with small number of leaders are not necessarily influential and manipulators may deliberately remove some leaders to improve their rank. In the followings we compare, through simulations and experiments, LeaderRank, PageRank and ranking by the number of fans.

### Comparison with Ranking by the Number of Fans

Ranking algorithms based on the network topology outperform ranking by merely the number of fans. We compare again user ranks with intrinsic qualities which are independent of the algorithm. One quantity which well characterizes the user influence is the number of times their collected bookmarks have been saved by the others. Though the leaders are not the only sources of bookmarks, influential users should still lead to wide spreading of their collected bookmarks. We denote the number of collected bookmarks by user 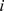 as 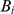 and the number of times these bookmarks are saved by others as 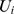. A user who recommends only high quality bookmarks should have a large value of 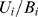.

**Figure 2:**
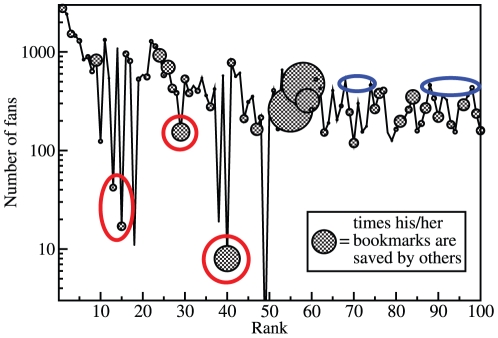
The number of fans of a user in descending order of the user rank by LeaderRank. The size of the solid circle is proportional to the value of 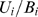, i.e. the average number of time their collected bookmarks are saved by others. Users highlighted with the red circles have a small number of fans but a large value of 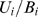. On the contrary, users highlighted with the blue circles have a large number of fans but a small value of 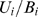.

We show in Fig. 2 the number of fans of a user in descending order of his/her rank by LeaderRank. The size of the circles is proportional to the value of 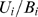. As we can see, there are users who are ranked high by LeaderRank but have only a small number of fans. Their ranks would greatly decrease if they are ranked by the number of fans. However, users highlighted with the red circles have relatively large 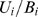 which shows that they are indeed high quality users. These users are identified by LeaderRank but not by the number of fans. On the contrary, there are users who have low rank but a large number of fans. The users highlighted with the blue circles have small 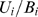 but a large number of fans. They are correctly ranked lower by LeaderRank.

**Figure 3:**
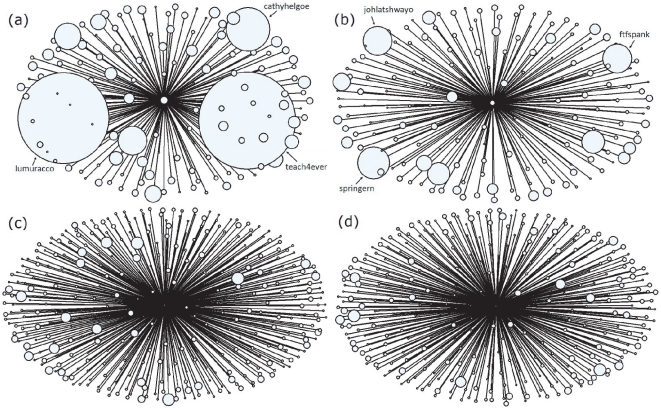
Users (a) *cffcoach*, (b) *pedersoj*, (c) *kanter* and (d) *britta*, who are ranked respectively at 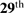, 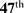, 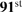 and 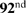 by LeaderRank, as surrounded by their fans. The size of circles represents the average number of times their collected bookmarks are saved by others.

To better understand these users, we draw in Fig. 3 particular examples of users with small number of fans but highly ranked, and users with a large number of fans but with a relatively low rank. As we can see in Figs. 3(a) and (b), users *cffcoach* and *pedersoj* are followed by fans with large values of 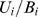, represented by the large size of circles. Though users *kanter* and *britta* have more fans, we can see from Figs. 3(c) and (d) that they are surrounded by much smaller circles. LeaderRank correctly gives them a lower rank, as compared to the ranking by merely the number of fans.

Similarly, just the leaders alone provides no absolute measure of influence, as it is the entire upstream connection to leaders which act as the information sources and contribute to the influence of a user. We show in Fig. S6 of *SI* that removing all the leaders may have a negative effect on the social influence of a user. All these results suggest that the leadership network is much more informative than simple ranking criteria such as the number of fans or leaders, and thus algorithms which well utilize the topology can provide a better ranking.

### Comparison with PageRank

In addition to identifying influential users, a good ranking algorithm for social networks should be tolerant of noisy data and robust against manipulations. These goals are better achieved by considering the collective ranking based on network topology. In the followings we compare the effectiveness and robustness between LeaderRank and PageRank, of which ranking is based on topology.

#### Effectiveness

How opinions spread and form in a community is an interesting question [13], [14]. To effectively spread opinion, one has to identify influential users and create an initial social inertia. For instance, companies may choose to start their adverts on influential leaders who are capable to initiate an extensive spreading through the Internet or SMS networks. Thus a smart algorithm which ranks influential users accurately is of great commercial values. On the other hand, effective ranking algorithm may serve its role to identify influential users for immunization and stop epidemic outbreak [15]. As an example, influential users who speed up junk mail spreading can be identified for targeted immunization. Here we show that LeaderRank is more capable than PageRank to identify influential users who initiate a *quicker* and *wider* spreading.

**Figure 4:**
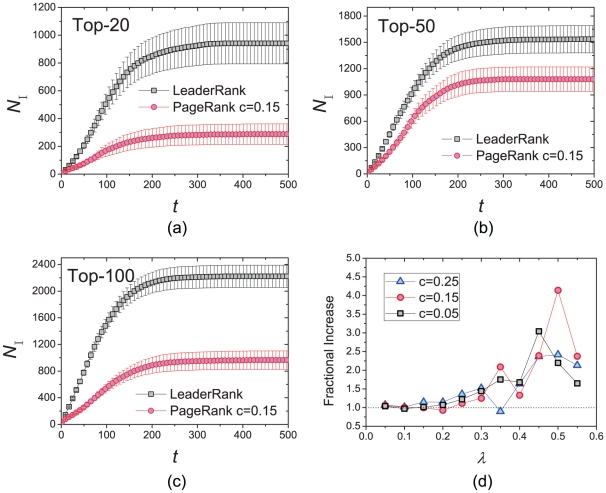
The cumulative number of infected users (including recovered users), 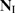, as a function of time, with initial infected to be the users either appear as (a) top-20, (b) top-50, and (c) top-100 by LeaderRank or PageRank (but not both). As we see from Table 1 in the top-20 case, the initial infected users by LeaderRank are *blackbeltjones*, *regina*, *zephoria* and *djakes*, while that by PageRank are *thetechguy*, *cffcoach*, *samoore* and *kevinrose*. Infection probability 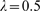 and return probability is set to 0.15 in PageRank. (d) As a function of 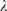, the quotient of the number of infected users in LeaderRank divided by that of PageRank, expressed as fractional increase.

Specifically, we employ a variant of the SIR model to examine the spreading influence of the top-ranked users [16]. At each step, from every infected individual, one randomly selected fan gets infected with probability 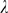, which resembles the direction of information flow. Infected individuals recover with probability 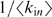 at each step, where 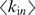 is the average in-degree of all users. To compare the ranking effectiveness, we set the initial infected to be the users either appear as the top 20 by LeaderRank or PageRank (but not both) in Table 1 , and compare the cumulative number of infected users (which includes infected and recovered users), denoted by 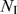, as a function of time. The initial infected users by the two algorithms are given in the caption of Fig. 4. This experiment resembles an opinion spreading initiated from the top users and observe how the opinion propagates. Figure 4(a) shows that infecting the top users from LeaderRank results in a faster growth and a higher saturated number of infected, indicating a *quicker* and *wider* spreading. To further confirm the effectiveness of LeaderRank, we also conduct experiments for the top 50 and top 100 ranked users either from LeaderRank or PageRank and obtain similar results which are shown in Figs. 4(b) and (c), respectively.

We show in Fig. 4 (d) the quotient of the total infected in LeaderRank divided by that of PageRank, with different infection probability 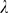. LeaderRank outperforms PageRank of various return probability and for a broad indicated range of 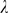. This reveals again a drawback of PageRank as the optimal return probability has to be found by extensive parameter tests. The results imply that spreading from both LeaderRank and PageRank users is limited when 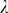 is small, but LeaderRank leads to a much wider opinion spreading when 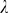 is large. For a virus outbreak, if intensive immunizations are implemented on the top ranked LeaderRank users, the final outbreak would be less extensive. All the above results show that LeaderRank is more effective than PageRank in identifying highly influential users, and is thus a better candidate for opinion spreading and to prevent a virus outbreak.

#### Tolerance of Noisy Data

Tolerance of ranking against spurious and missing links, i.e. false positive and false negative connections, is crucial when network structure is subject to noisy observations [17]. Social network data may be unreliable, especially when users are required to explicitly indicate relationship with others [18]. It is like, to state whether neighbors are friends if they just greet each other when they meet. The same happens for networks other than social networks but with a rather different cause. For example, protein connections obtained from biological experiments often include numerous false positives and false negatives [19]. Other than ambiguous personal relationship, it is also costly and technically difficult to explore social networks comprehensively. Efforts have thus been made to predict the missing connections [20] and on such noisy networks, we should develop ranking algorithms which are tolerant of spurious and missing links.

**Figure 5:**
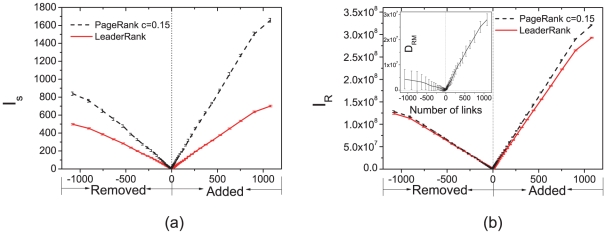
The impact on (a) scores and (b) ranking as a function of number of links added and removed. Inset: (b) the difference in ranking mobility between LeaderRank and PageRank.

To examine the tolerance of LeaderRank and PageRank against noisy data, we measure the change in scores and rankings when links are added or removed randomly. These links correspond to the spurious or missing relationship among leaders and fans. The scores obtained from the modified graph are compared to those from the original graph, by measuring the impact 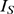 on score, as given by
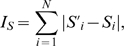
(4) and 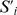 correspond to the scores obtained respectively from the original and modified graph. We measure 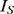 for both LeaderRank and PageRank subject to the same modifications. As shown in Fig. 5 (a), 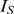 increases with the number of links added or removed. Remarkably, much smaller values of 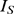 are obtained from LeaderRank when compared to PageRank, regardless of the addition or removal of links. In a word, LeaderRank is more tolerant than PageRank against noisy topology, and thus has a high potential in applications on noisy social networks or protein-protein networks [21].

Since a small change in scores in LeaderRank may not directly correspond to a small change in ranking, we define a similar measure to examine the impact 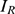 on ranking, given by
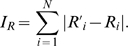
(5)As shown in Fig. 5 (b), a smaller difference between 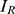 of LeaderRank and PageRank is observed as compared to 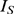. Nevertheless 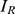 of LeaderRank is smaller, as shown by 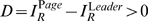 in the inset. Once again, these observations in 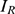 suggest that LeaderRank is more tolerant of noise in topology and hence a better candidate for ranking in noisy networks.

#### Robustness against Spammers

Malicious activities are common in social networks, in particular when users manipulate to gain skewed reputation [10]. One example of manipulation is called *Sybil Attack* [22], in which spammers deliberately create fake entities to obtain disproportionately high rank. The problems become intolerable if this manipulation causes recommendation of bad commodities or biased opinion in social networks. In WWW, there are also stories of companies manipulating Google search engine to obtain higher ranks in search results [23]. To cope with this loophole, we show that LeaderRank is more robust than PageRank against this type of attacks.

**Figure 6:**
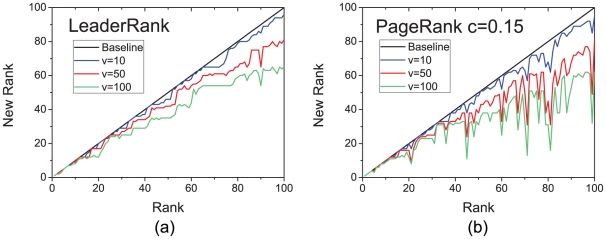
The manipulated rank as obtained by (a) LeaderRank and (b) PageRank, after the addition of 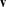 **fake fans, with** 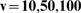 **.**

Specifically, we simulate the situation where a user creates 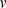 fake fans, and compare the ranking robustness in LeaderRank and PageRank. The horizontal axis of Figs. 6(a) and (b) shows respectively for LeaderRank and PageRank the original rank of a user, and the vertical axis shows his/her manipulated rank after the addition of 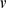 fake fans. Vertical downward shift from the dashed diagonal corresponds to the increase in rankings, and thus a successful manipulation. As we can see, LeaderRank is more robust against spammers as the change of rankings is much smaller than that by PageRank. These results show that LeaderRank is a better candidate for robust rankings against manipulations.

### Experiment

To let readers better understand social influences as quantified by LeaderRank, we established a webpage http://rank.sesamr.com which uses LeaderRank to rank users in delicious.com. By providing their username, delicious users can easily obtain their rank and other information including the influence of leaders and fans. Users can also examine the change of their influence when they have new leaders and fans. For instance, the user *babyann519* had a low rank of 607512 before six other users found her important bookmarks and added her as a leader. She now has a rank of 99440, a much higher rank which shows the increase in her influence.

## Discussion

After going through the above details, we may conclude that identifying influential users is not a simple task. It is not merely answering who is the best, but as well to consider the influences and consequences brought by a ranking algorithm. These consequences are of particular importance for social networks, which are fundamentally different from networks of webpages. For instance, the ranking should be robust against noisy data and smart manipulations. This leads us to answer a much broader question by devising a robust and generic algorithm, than merely identifying the leaders.

We suggest that LeaderRank may serve as a prototype of ranking algorithms applicable to rank users in social networks. As personal relationships are quickly evolving, the adaptive and parameter-free nature of LeaderRank eliminates the need of frequent calibration. In addition, this simple algorithm outperforms PageRank in several important aspects. In this paper, we see that LeaderRank identifies users who lead to quick and extensive spreading of opinions. This is important for online applications which feature information spreading. On the other hand, LeaderRank is tolerant of spurious and missing links, which benefits applications with noisy data, especially personal relationship. To deal with ranking loopholes, LeaderRank is robust against manipulations. These results make LeaderRank a good candidate for ranking users as well as other ranking tasks.

Though LeaderRank is already an effective algorithm, extensions may lead to further improvement. For instance, the role of the ground node would be more prominent if weights are set on the in- and out-links to each node, according to its significance or other criteria. In cases where users can be characterized by specific categories such as interests, multiple ground nodes with different category can be introduced, and links between users and ground node in the same category are assigned with higher weights. This formulation facilitates the probability flow between users in the same categories, and may identify influential users in each category. Such potential application would require further investigations. Other than ranking users, LeaderRank can also be generalized to applications ranging from blog plagiarizer identification [24], to stopping species lost in ecosystem [25]. These simple modifications may lead to substanial improvements in performance.

Identifying influential users in social networks is still a task on which we may overlook. As accompanied by the expanding popularity of online communities, leader identification may reinforce their development. This further facilitates collective search through online communities and may one day complement the current search paradigm. For sure in the near future, technological advance will provide more information to quantify user influence, but at the same time will scale up the network size and make ranking tasks more challenging. LeaderRank suggested here may serve as a potential candidate to face this challenge and well utilize the power of social influences.

## Supporting Information

Figure S1The score flow from a node to (a) the ground node in LeaderRank and (b) random nodes in PageRank as a function of 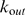, the number of leaders.(EPS)

Figure S2The ratio of saved bookmarks to the number of leaders as a function of 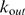.(EPS)

Figure S3 The score as a function of rank obtained from the LeaderRank, PageRank and ranking by the number of fans. Zipf's law is observed for these algorithms.
(EPS)

Figure S4The overlap between LeaderRank and PageRank, and LeaderRank and ranking by the number of fans, as well as PageRank and ranking by the number of fans, for the top-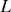 users.(EPS)

Figure S5The average number of leaders of the top-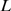 users as ranked by LeaderRank and PageRank. Inset: the average number of leaders against the logarithm of 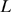.(EPS)

Figure S6 The rank of a user after removing all his/her leaders, as compared to his/her original rank as obtained by (a) LeaderRank and (b) PageRank. The black solid line corresponds to the equality of the new and original rank.
(EPS)

Table S1 Top 100 users ranked by LeaderRank, PageRank and the number of fans.
(PDF)

Text S1 Brief discussion of the results in the figures of \emph{SI} and the source code of LeaderRank algorithm.
(PDF)

## References

[1] Lampe C, Ellison N, Steinfield C (2006). A Face(book) in the crowd: social searching vs. social browsing..

[2] Vieira MV, Fonseca BM, Damazio R, Golgher PB, de Castro Reis D (2007). Efficient search ranking in social networks..

[3] Easley D, Kleinberg J (2010). Networks, Crowds and Markets..

[4] Kleinberg J (1999). Authoritative sources in a hyperlinked environment.. J ACM.

[5] Park J, Newman MEJ (2005). A network-based ranking system for US college football.. J Stat Mech.

[6] Radicchi F, Fortunato S, Markines B, Vespignani A (2009). Diffusion of scientific credits and the ranking of scientists.. Phys Rev E.

[7] Chen P, Xie H, Maslov S, Redner S (2007). Finding scientific gems with Google.. J Inform.

[8] Page L, Brin S, Motwani R, Winograd T (1999). The PageRank citation ranking: Bringing order to the web.. Technical Report Stanford InfoLab.

[9] Brin S, Page L (1998). The anatomy of a large-scale hypertextual web search engine.. Comput Networks and ISDN Systems.

[10] Masum H, Zhang YC (2004). Manifesto for the reputation society.. First Monday.

[11] Watts DJ, Strogatz SH (1998). Collective dynamics of ‘small world’ networks.. Nature.

[12] Newman MEJ (2002). Assortative mixing in networks.. Phys Rev Lett.

[13] Castellano C, Fortunato S, Loreto V (2009). Statistical physics of social dynamics.. Rev Mod Phys.

[14] Galam S (2002). Minority opinion spreading in random geometry.. Eur Phys J B.

[15] Pastor-Satorras R, Vespignani A (2002). Immunization of complex networks.. Phys Rev E.

[16] Yang R, Wang BH, Ren J, Bai WJ, Shi ZW (2007). Epidemic spreading on heterogeneous networks with identical infectivity.. Phys Lett A.

[17] Guimerá R, Sales-Pardo M (2009). Missing and spurious interactions and the reconstruction of complex networks.. Proc Natl Acad Sci USA.

[18] Marsden PV (1990). Network data and measurement.. Annual Review of Sociology.

[19] Legrain P, Wojcik J, Gauthier JM (2001). Protein–protein interaction maps: a lead towards cellular functions.. Trends in Genetics.

[20] Lü L, Zhou T (2011). Link prediction in complex networks: A survey.. Physica A.

[21] Chen J, Aronow BJ, Jegga AG (2009). Disease candidate gene identification and prioritization using protein interaction networks,. BMC Bioinformatics.

[22] Douceur JR (2002). The Sybil Attack..

[23] Levine BN, Shields C, Margolin BN (2006). A survey of solutions to the sybil attack.. Technical Report of Univ of Massachussets Amherst.

[24] Gayo-Avello D (2010). Nepotistic relationships in Twitter and their impact on rank prestige algorithms.. http://arxiv.org.

[25] Allesina S, Pascual M (2009). Googling food webs: Can an eigenvector measure species' importance for coextinctions?. PLoS Comput Bio.

