# Supplementary material for: Leaders in Social Networks, the Delicious Case
Source: PLoS One. 2011 Jun 27;6(6):e21202. doi: 10.1371/journal.pone.0021202 (PMC3124485; doi:10.1371/journal.pone.0021202)
Supplement: Table S1 — Top 100 users ranked by LeaderRank, PageRank and the number of fans. (PDF) [file pone.0021202.s007.pdf]

Table S1: Top 100 users ranked by LeaderRank, PageRank and the number of fans.

Rank	LeaderRank		PageRank (c=0.15)		Number of fans	
	User ID	Score	User ID	Score	User ID	Fans #
1	adobe	452	adobe	808	adobe	2768
2	twit	382	twit	726	twit	2422
3	wfryer	369	twitarmy	629	wfryer	1528
4	willrich	358	thetechguy	536	willrich	1466
5	joshua	264	cffcoach	529	merlinmann	1326
6	cshirky	234	wfryer	492	joshua	1296
7	hrheingold	217	willrich	475	steverubel	1284
8	ewan.mcintosh	214	joshua	375	jgwalls	1142
9	dwarlick	202	jdehaan	337	regine	1086
10	twitarmy	200	lseymour	334	jonhicks	956
11	merlinmann	186	isola	315	kevinrose	924
12	blackbeltjones	171	cshirky	294	hrheingold	894
13	jdehaan	170	secondlife	291	cshirky	837
14	regine	170	ewan.mcintosh	288	dwarlick	827
15	lseymour	168	hrheingold	285	zephoria	812
16	jonhicks	168	merlinmann	267	ambermac	781
17	zephoria	159	jonhicks	262	jgates513	702
18	isola	159	samoore	261	ramitsethi	660
19	djakes	158	dwarlick	261	ewan.mcintosh	635
20	secondlife	156	kevinrose	256	cory_arcangel	613
21	edtechtalk	152	iwantsandy	249	secondlife	587
22	steverubel	150	regine	248	brightideasguru	586
23	jgwalls	142	jgwalls	234	judell	576
24	kevinrose	135	steverubel	222	warrenellis	566
25	brightideasguru	124	edtechtalk	214	edtechtalk	559
26	jgates513	123	zephoria	212	elisebauer	545
27	cogdog	120	nichoson	210	blackbeltjones	541
28	joi_ito	119	djakes	206	hokie62798	533
29	cffcoach	114	blackbeltjones	206	djakes	531
30	hokie62798	113	elisebauer	203	infosthetics	527
31	samoore	112	dr.coop	178	bibliodyssey	509
32	cityofsound	112	sdigrego	172	jakkarin	476
33	heyjude	110	ambermac	161	chrisbrogan	474
34	elisebauer	108	ureerat	160	russelldavies	461
35	veen	104	jgates513	160	makemagazine	461
36	shareski	102	glass	160	ericerb	455
37	mathowie	101	brightideasguru	159	cityofsound	454
38	thetechguy	101	ramitsethi	150	jummumboy	435
39	judell	100	hokie62798	150	jdawg	433
40	nichoson	100	cogdog	148	earlysound	430
41	ambermac	99	joi_ito	146	jzawodn	429
42	warrenellis	96	heyjude	145	cogdog	428
43	cory_arcangel	93	judell	143	mathowie	421
44	jutecht	92	cityofsound	142	plasticbag	407
45	tomc	92	kawid	141	fredwilson	407
46	choconancy	92	ceonyc	140	shanselman	406
47	pedersoj	91	jdawg	139	heyjude	405
48	mamamusings	91	bearsgonewild	136	leolaporte	404
49	sdigrego	91	warrenellis	136	joi_ito	385

Rank	LeaderRank		PageRank (c=0.15)		Number of fans	
	User ID	Score	User ID	Score	User ID	Fans #
50	linkorama	90	benchaporn	134	samoore	384
51	plasticbag	90	veen	130	curson12005	381
52	sebpaquet	88	shareski	129	miyagawa	364
53	ramitsethi	87	mathowie	127	veen	363
54	snbeach50	83	choconancy	126	tuckermax	363
55	ureerat	81	shanselman	126	kanter	359
56	jdawg	81	jutecht	126	choconancy	354
57	teach42	79	linkorama	124	deusx	351
58	jakkarin	78	kick_out_the_internet_jams	123	aengle	351
59	benchaporn	78	cory_arcangel	123	lomo	350
60	budtheteacher	77	selmav	121	bren	344
61	infosthetics	75	pedersoj	119	wearehugh	342
62	jzawodn	75	fju_web20	114	53os	342
63	raelity	73	mamamusings	113	101cookbooks	340
64	chrisdodo	72	tomc	113	ginatrapani	336
65	fredwilson	70	sebpaquet	111	angusf	333
66	timo	70	bibliodyssey	111	zheng	331
67	elemenous	69	apluscert	111	megsie	331
68	bibliodyssey	69	alexdroege	109	britta	327
69	iteachdigital	69	plasticbag	109	benchaporn	321
70	timlauer	69	madro	108	teach42	319
71	fstutzman	69	lisalis	108	knowhow	312
72	foe	69	fredwilson	106	tomc	312
73	migurski	69	infosthetics	105	snbeach50	307
74	russelldavies	68	williams_jeff	104	marisaolson	305
75	alexdroege	67	101cookbooks	104	fstutzman	301
76	curson12005	66	cablack	104	edans	300
77	shanselman	65	snbeach50	103	jasonmcalacanis	298
78	twitter_edtech	65	jzawodn	103	williams_jeff	292
79	kick_out_the_internet_jams	64	wswu	103	yugop	290
80	msippey	63	davepro14	102	wang1	290
81	qdsouza	62	pamanapa	100	dhinchcliffe	288
82	anne	62	fju_webfund	100	ani625	288
83	brasst	62	teach42	99	music	287
84	aengle	61	tarisamatsumoto	98	elemenous	284
85	ceonyc	61	fju_univintro	96	toxi	282
86	kfisch	61	russelldavies	95	google	281
87	ehubbell	60	makemagazine	95	shareski	278
88	makemagazine	60	fju_inetcomp	95	mbauwens	275
89	101cookbooks	59	clydekmann	93	design	275
90	dr.coop	58	atrusty	92	mediaeater	274
91	kanter	58	budtheteacher	92	ehubbell	271
92	britta	58	elemenous	91	imao	270
93	courosa	58	fstutzman	90	ureerat_wat	267
94	mguhlin	57	twitter_edtech	90	ma.la	265
95	marisaolson	56	curson12005	90	alexdroege	265
96	williams_jeff	56	timo	89	jewel_lee27	264
97	tuckermax	56	raelity	89	linkorama	262
98	jummumboy	56	iteachdigital	89	raganwald	261
99	district6	56	shiang	88	brasst	261
100	chrislehmann	55	knowhow	87	budtheteacher	260
