# Supplementary material for: Leaders in Social Networks, the Delicious Case
Source: PLoS One. 2011 Jun 27;6(6):e21202. doi: 10.1371/journal.pone.0021202 (PMC3124485; doi:10.1371/journal.pone.0021202)
Supplement: Text S1 — Brief discussion of the results in the figures of \emph{SI} and the source code of LeaderRank algorithm. (PDF) [file pone.0021202.s008.pdf]

Supporting Information

1. Primitivity and Convergence

We first show that the stochastic matrix P is primitive by showing P^6 is positive, i.e. the elements in P^6 are all greater than zero. It is equivalent to show that any pair of nodes are connected in exactly 6 steps (6 hops). For nodes with at least one link, ground node guarantees the co-existence of loops of size 2 and 3. Starting at any node with 2 loops of size 2 and a path through the ground node, we can reach any other node (excluding the ground node but including itself) in exactly 6 steps. To reach the ground node in exactly 6 steps, we make use of one loop of size 3 and one loop of size 2 before hopping to the ground node. The same is true to reach the other nodes from the ground node.

As P is a right stochastic matrix, the transpose P^T would be the usual transition matrix by conventional matrix multiplication, such that $\vec{s}(t_c) = P^T\vec{s}(t_c)$. We then show that 1 is an eigenvalue of P, and thus of P^T. The matrix P, which is row-normalized, has obviously an eigenvalue 1 with eigenvector filled with all equal entries, and thus 1 is an eigenvalue of P. To show the uniqueness of eigenvector associated with eigenvalue 1, we assume that there exists another eigenvector $\vec{v}$ for eigenvalue 1 with heterogeneous entries. Let v_j to be the entry of this eigenvector with $|v_j| > |v_i|$ for all i. We then choose the eigenvector such that v_j is positive. As P is primitive, we consider a matrix P^m where all entries are positive. The assumption of eigenvector with heterogeneous entries leads to the following contradiction

$$\vec{v} = P^m \cdot \vec{v} \Rightarrow v_j = \sum_i p'_{ij} v_i < \sum_i p'_{ij} v_j = v_j, \tag{1}$$

where p' denotes the elements of P^m. The contradiction implies that for P^m, and hence P, the eigenvector with heterogeneous entries does not exist for eigenvalue 1, and thus P^T has an unique eigenvector associated with eigenvalue 1, i.e. a unique steady state.

2. Differences between LeaderRank and PageRank

The obvious difference between LeaderRank and PageRank lies in the formulation, where the ground node in LeaderRank plays an important role in regulating probability flows, making LeaderRank a parameter-free algorithm. An essential difference does lie in the heart of dynamics. In LeaderRank, the score flow from node i to the ground node is given by

$$f_{i\to g} = \frac{s_i(t_c)}{k_i^{\rm out}}, \tag{2}$$

while in PageRank the score flow from node i to a random node is given by

$$f_{i\to {\rm rand}} = cs_i(t_c), \tag{3}$$

where c is the return probability. As shown in Fig. S1, $f_{i\to g}$ in LeaderRank is inversely proportional to the out-degree of i, i.e. the number of leaders of i, as expected from the above equation. On the other hand, $f_{i\to {\rm rand}}$ in PageRank show no obvious trend with the number of leaders. Such observation corresponds to a fundamental difference between LeaderRank and PageRank.

We may interpret the physical reasons in the following examples. In social networks, the score donated to the ground node can be interpreted as the information obtained from random browsing, in contrast to the ordinary way of information acquisition from leaders. The ground node can thus be considered as a centralized leader who provides general information. We argue that fans who have a large number of leaders may acquire less information from each leader, including this centralized leader, leading to the relation in Fig. S1(a). Similar relation is observed in our empirical analyses with delicious data in Fig. S2, which show that the ratio of saved bookmarks to the number of leader, decreases with $k_{\rm out}$ of the user. The same deduction can be obtained

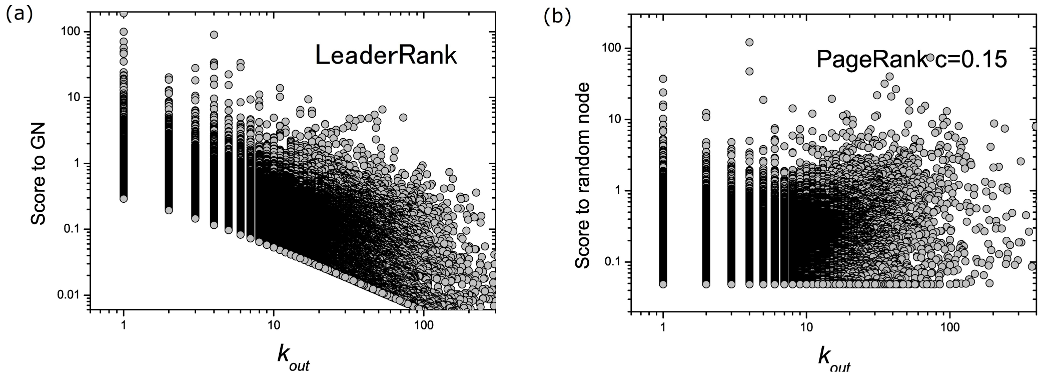

Fig. S1: The score flow from a node to (a) the ground node in LeaderRank and (b) random nodes in PageRank as a function of k_{out}, the number of leaders.

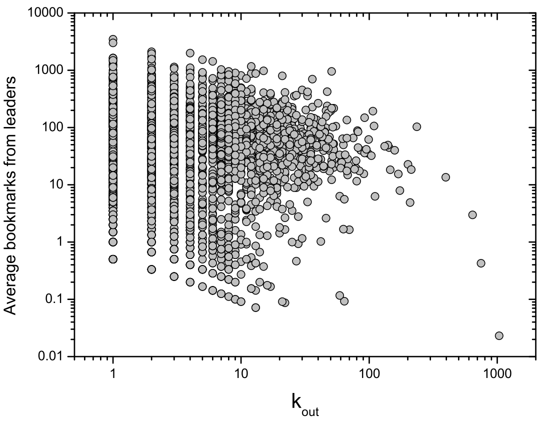

Fig. S2: The ratio of saved bookmarks to the number of leaders as a function of k_{out}.

from the point of view of leaders. If we assume that the average number of bookmarks provided by each leader is not indefinitely different, nodes with small number of leaders receive only little information from leaders and thus they have to acquire more information from the ground node.

In terms of ranking, users with few leaders should have small voting rights for leaders, otherwise they may produce a strong bias if they donate all their score to only one or two leaders. LeaderRank, from which a negative correlation is introduced between score flow to leaders and out-degree (i.e. flow to leaders is smaller from users with smaller out-degree), would lead to a better ranking when compared to PageRank.

As the last example, web surfers surfing on websites with small out-degree have limited choices of hyperlink and by higher chance jump to another random website. On the contrary, web surfers are more likely to go through hyperlinks if there are lots of them on the website. Such cases correspond to a small flow from nodes with large k_{out} to the ground node, which is captured by LeaderRank.

3. The top-100 ranked users

Here we report the top 100 ranked users and their corresponding scores as obtained by LeaderRank, PageRank and the number of fans. As one unit of score is initialized on every node in

LeaderRank and PageRank, the scores sum up to N in these two rankings. The last two columns show the top-100 users with the largest number of fans, and their corresponding number of fans.

Table S1: Top 100 users ranked by LeaderRank, PageRank and the number of fans.

Rank	LeaderRank		PageRank (c=0.15)		Number of fans	
	User ID	Score	User ID	Score	User ID	Fans #
1	adobe	452	adobe	808	adobe	2768
2	twit	382	twit	726	twit	2422
3	wfryer	369	twitarmy	629	wfryer	1528
4	willrich	358	thetechguy	536	willrich	1466
5	joshua	264	cffcoach	529	merlinmann	1326
6	cshirky	234	wfryer	492	joshua	1296
7	hrheingold	217	willrich	475	steverubel	1284
8	ewan.mcintosh	214	joshua	375	jgwalls	1142
9	dwarlick	202	jdehaan	337	regine	1086
10	twitarmy	200	lseymour	334	jonhicks	956
11	merlinmann	186	isola	315	kevinrose	924
12	blackbeltjones	171	cshirky	294	hrheingold	894
13	jdehaan	170	secondlife	291	cshirky	837
14	regine	170	ewan.mcintosh	288	dwarlick	827
15	lseymour	168	hrheingold	285	zephoria	812
16	jonhicks	168	merlinmann	267	ambermac	781
17	zephoria	159	jonhicks	262	jgates513	702
18	isola	159	samoore	261	ramitsethi	660
19	djakes	158	dwarlick	261	ewan.mcintosh	635
20	secondlife	156	kevinrose	256	cory_arcangel	613
21	edtechtalk	152	iwantsandy	249	secondlife	587
22	steverubel	150	regine	248	brightideasguru	586
23	jgwalls	142	jgwalls	234	judell	576
24	kevinrose	135	steverubel	222	warrenellis	566
25	brightideasguru	124	edtechtalk	214	edtechtalk	559
26	jgates513	123	zephoria	212	elisebauer	545
27	cogdog	120	nichoson	210	blackbeltjones	541
28	joi_ito	119	djakes	206	hokie62798	533
29	cffcoach	114	blackbeltjones	206	djakes	531
30	hokie62798	113	elisebauer	203	infosthetics	527
31	samoore	112	dr.coop	178	bibliodyssey	509
32	cityofsound	112	sdigrego	172	jakkarin	476
33	heyjude	110	ambermac	161	chrisbrogan	474
34	elisebauer	108	ureerat	160	russelldavies	461
35	veen	104	jgates513	160	makemagazine	461
36	shareski	102	glass	160	ericerb	455
37	mathowie	101	brightideasguru	159	cityofsound	454
38	thetechguy	101	ramitsethi	150	jummumboy	435
39	judell	100	hokie62798	150	jdawg	433
40	nichoson	100	cogdog	148	earlysound	430
41	ambermac	99	joi_ito	146	jzawodn	429
42	warrenellis	96	heyjude	145	cogdog	428
43	cory_arcangel	93	judell	143	mathowie	421
44	jutecht	92	cityofsound	142	plasticbag	407
45	tomc	92	kawid	141	fredwilson	407
46	choconancy	92	ceonyc	140	shanselman	406
47	pedersoj	91	jdawg	139	heyjude	405

Rank	LeaderRank		PageRank (c=0.15)		Number of fans	
	User ID	Score	User ID	Score	User ID	Fans #
48	mamamusings	91	bearsgonewild	136	leolaporte	404
49	sdigrego	91	warrenellis	136	joi_ito	385
50	linkorama	90	benchaporn	134	samoore	384
51	plasticbag	90	veen	130	curson12005	381
52	sebpaquet	88	shareski	129	miyagawa	364
53	ramitsethi	87	mathowie	127	veen	363
54	snbeach50	83	choconancy	126	tuckermax	363
55	ureerat	81	shanselman	126	kanter	359
56	jdawg	81	jutecht	126	choconancy	354
57	teach42	79	linkorama	124	deusx	351
58	jakkarin	78	kick_out_the_internet_jams	123	aengle	351
59	benchaporn	78	cory_arcangel	123	lomo	350
60	budtheteacher	77	selmav	121	bren	344
61	infosthetics	75	pedersoj	119	wearehugh	342
62	jzawodn	75	fju_web20	114	53os	342
63	raelity	73	mamamusings	113	101cookbooks	340
64	chrisdodo	72	tomc	113	ginatrapani	336
65	fredwilson	70	sebpaquet	111	angusf	333
66	timo	70	bibliodyssey	111	zheng	331
67	elemenous	69	apluscert	111	megsie	331
68	bibliodyssey	69	alexdroege	109	britta	327
69	iteachdigital	69	plasticbag	109	benchaporn	321
70	timlauer	69	madro	108	teach42	319
71	fstutzman	69	lisalis	108	knowhow	312
72	foe	69	fredwilson	106	tomc	312
73	migurski	69	infosthetics	105	snbeach50	307
74	russelldavies	68	williams_jeff	104	marisaolson	305
75	alexdroege	67	101cookbooks	104	fstutzman	301
76	curson12005	66	cablack	104	edans	300
77	shanselman	65	snbeach50	103	jasonmcalacanis	298
78	twitter_edtech	65	jzawodn	103	williams_jeff	292
79	kick_out_the_internet_jams	64	wswu	103	yugop	290
80	msippey	63	davepro14	102	wang1	290
81	qdsouza	62	pamanapa	100	dhinchcliffe	288
82	anne	62	fju_webfund	100	ani625	288
83	brasst	62	teach42	99	music	287
84	aengle	61	tarisamatsumoto	98	elemenous	284
85	ceonyc	61	fju_univintro	96	toxi	282
86	kfisch	61	russelldavies	95	google	281
87	ehubbell	60	makemagazine	95	shareski	278
88	makemagazine	60	fju_inetcomp	95	mbauwens	275
89	101cookbooks	59	clydekmann	93	design	275
90	dr.coop	58	atrusty	92	mediaeater	274
91	kanter	58	budtheteacher	92	ehubbell	271
92	britta	58	elemenous	91	imao	270
93	courosa	58	fstutzman	90	ureerat_wat	267
94	mguhlin	57	twitter_edtech	90	ma.la	265
95	marisaolson	56	curson12005	90	alexdroege	265
96	williams_jeff	56	timo	89	jewel_lee27	264
97	tuckermax	56	raelity	89	linkorama	262
98	jummumboy	56	iteachdigital	89	raganwald	261
99	district6	56	shiang	88	brasst	261

Rank	LeaderRank		PageRank (c=0.15)		Number of fans	
	User ID	Score	User ID	Score	User ID	Fans #
100	chrislehmann	55	knowhow	87	budtheteacher	260

4. Zipf's law

As shown in Fig. S3, Zipfs law is observed for all the three ranking algorithms. We plot the score of each user against his/her rank and observe a power-law decaying. Notice that, although similar relation between score and rank is observed among the three algorithms, the ranking of individual is different by different algorithms

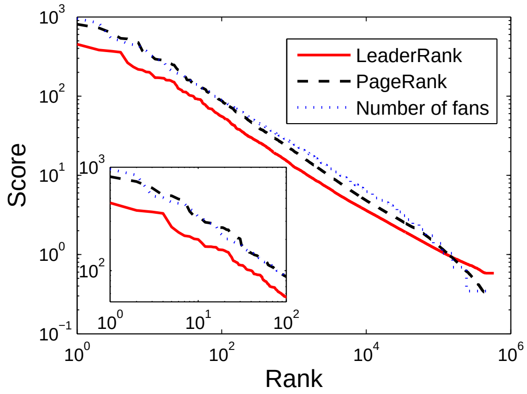

Fig. S3: The score as a function of rank obtained from the LeaderRank, PageRank and ranking by the number of fans. Zipf's law is observed for these algorithms.

5. Comparisons among ranking results from different ranking algorithms

We show in Fig. S4 the overlap of ranking between LeaderRank and PageRank, as well as LeaderRank and the number of fans. We plot as well the overlap between PageRank and the number of fans for reference. These results show that LeaderRank is closer to PageRank, than merely ranking by the number of fans, and both LeaderRank and PageRank show positive correlation with the number of fans. Though rankings from LeaderRank and PageRank seems to have large overlap, the rankings of individual are different, as can be seen in Table S1. As shown in Fig. S5, average number of leaders of the top users as ranked by PageRank is always smaller than that by LeaderRank. It implies that PageRank tends to assign high rank to nodes with small number of leaders, which is unfair to nodes with large number of leaders. We emphasize again individual rankings are different though the shape of the curves form LeaderRank and PageRank looks similar.

6. Negative effect by removal of leaders

We show in Fig. S6 that there is a negative effect in the rank of a user by removing all his/her leaders. As we can see for both LeaderRank and PageRank, many users are lower in rank after removing their leaders. These results suggest that considering just the leaders alone provides no absolute measure of influence, as removing the entire upstream connection to leaders user may have a negative effect on the social influence of an influential user. In other words, we have to consider the entire upstream topology to quantify the social influence of a user.

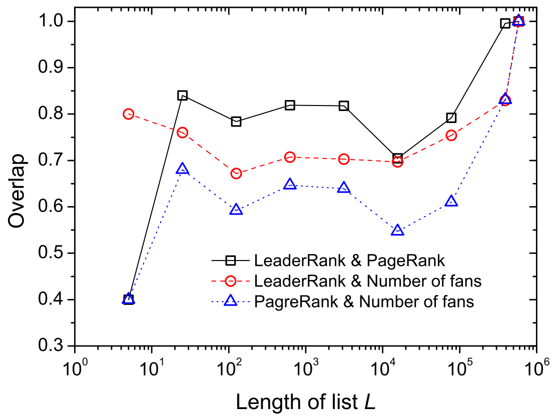

Fig. S4: The overlap between LeaderRank and PageRank, and LeaderRank and ranking by the number of fans, as well as PageRank and ranking by the number of fans, for the top-L users.

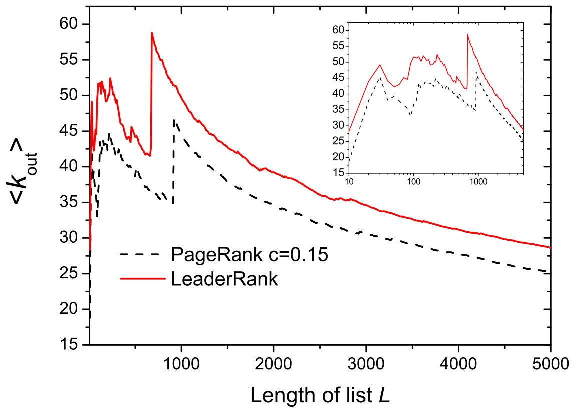

Fig. S5: The average number of leaders of the top-L users as ranked by LeaderRank and PageRank. Inset: the average number of leaders against the logarithm of L.

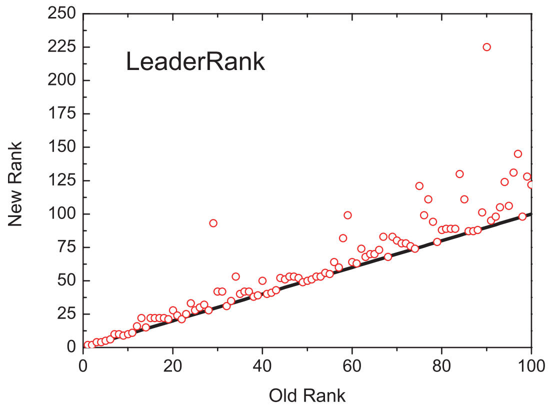

(a)

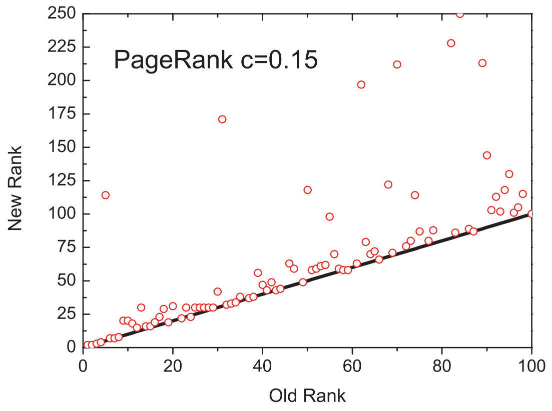

(b)

Fig. S6: The rank of a user after removing all his/her leaders, as compared to his/her original rank as obtained by (a) LeaderRank and (b) PageRank. The black solid line corresponds to the equality of the new and original rank.

7. Source Code for the LeaderRank algorithm

We attached here the source code for running LeaderRank algorithm:

```
% This is a Matlab M-file for LeaderRank.
E=load('Network.dat'); % Read the network data with different pairs
                       % of 'fan leader' in consecutive rows, and the
                       % labels of nodes should start from 1
l=length(E);           % l is the number of links
N=max(max(E));         % N is the number of nodes.

% Add ground node and create adjacency matrix P
   EG1=zeros(N,2);
   EG2=zeros(N,2);
   for i=1:N
      EG1(i,1)=N+1;
      EG1(i,2)=i;
   end
   EG2(:,1)=EG1(:,2);
   EG2(:,2)=EG1(:,1);
   E=[E;EG1;EG2];
   P=sparse(E(:,1),E(:,2),1);
   D_in=sum(P);       % in degree
   D_out=sum(P');     % out degree

% Transition matrix PP
   EE=zeros(N+1,2);
   for j=1:N+1
      EE(j,1)=j;
      EE(j,2)=1/D_out(j);
   end
   D=sparse(EE(:,1),EE(:,1),EE(:,2));
   PP=D*P;

% Diffusion to stable state.
   God=zeros(N+1,1);
   God(1:N,1)=1;                 % Assign initial resource
   error=10000;                  % error is the average error of nodes' scores.
   error_threshold=0.00002;      % It is a tunable parameter controlling the
                                 % error tolerance.
   step=1;
   while error>error_threshold
       step
       M=God;
       God=PP'*God;
       error=sum(abs(God-M)./M)/(N+1);
       step=step+1;
   end
   b=God(N+1)/N;
   God=God+b;
   God(N+1)=0;

% Write the ranking results to "Results.dat": node's ID & Score
   R=zeros(N,2);
   R(:,1)=[1:N]';
```

```
R(:,2)=God(1:N);
[ b, pos ] = sort( -R( :, 2 ));
R = R(pos,: );
fid = fopen('Results.dat','w');
for i=1:N
    fprintf(fid,'%d %f \n',R(i,1),R(i,2));
end
fclose(fid);
```
